# 

**Published:** 1888-04

**Authors:** 


Death of Dr. Wm. Glover.—Dr. Wm. Glover, late of Cedar Creek, Bastrop county, Texas, died of congestion of the lungs at Lovelady, Texas, on the 20th of March ult. Dr. Glover had left his home recently in hope of benefiting his health, which, for some time, had been failing. He was a member in good standing in the Physicians' Mutual Benefit Association of Texas, and assessment (No. 6) has been issued for the benefit of his family. He was holder or certificate No. 105.

DOCTOR'S SON MARRIED.—Mr. Montrose Burt, eldest son of the late popular Secretary of the Texas State Medical Association, Dr. W. J. Burt, was married at Austin on the 4th inst., to Miss Leontine Brunet.

